# Stain Susceptibility of Composite Resins: Pigment Penetration Analysis

**DOI:** 10.3390/ma15144874

**Published:** 2022-07-13

**Authors:** Francesca Cinelli, Daniele Scaminaci Russo, Michele Nieri, Luca Giachetti

**Affiliations:** Unit of Dentistry, Department of Experimental and Clinical Medicine, University of Florence, Via del Ponte di Mezzo, 48-50127 Firenze, Italy; francescacinelli05@gmail.com (F.C.); daniele.scaminacirusso@unifi.it (D.S.R.); michelenieri@gmail.com (M.N.)

**Keywords:** composite resin, staining, color stability, discoloration, stain penetration

## Abstract

Composite resins are considered the material of choice for esthetic direct restorations, considering both their satisfying esthetic and mechanical properties. The success of composite resin restorations depends highly on their color stability. Discoloration causes color mismatch, consequent patient dissatisfaction, and eventually additional costs for correction/replacement of the restoration. The purpose of this study was to evaluate the degree of pigment penetration within the composite resins, in order to understand how discoloration can be treated properly. Two different commercially available composite resins were compared in the study: a nano-filled composite resin and a non-homogeneous micro-hybrid composite resin. A coffee solution was used to induce staining of the materials. Subsequently, the penetration of the pigments was measured by analyzing the color from the outside to the inside of the specimen. 14 levels were analyzed starting from 0.1 mm to 3.0 mm in depth. The ANOVA test demonstrated a statistically significant difference (*p* < 0.0001) between test and control groups up to a depth of 1.0 mm for the nano-filled composite and up to a depth of 2.0 mm for the non-homogeneous micro-hybrid composite. The two composite resin materials, subjected to pigmenting treatment, underwent a color variation with different patterns.

## 1. Introduction

Modern dentistry is required to deal with the increased demand for highly aesthetic restorations, in which the color match is consequently a factor of primary importance. Composite resins are considered the material of choice for esthetic direct restorations in both anterior and posterior teeth. This is due to the fact that these materials exhibit both satisfying esthetic and mechanical properties, allowing for a minimally invasive approach. The success of composite resin restorations depends highly on their color stability and on the finishing and polishing of their surface. In fact, discoloration and marginal stains are one of the most common causes of failure in composite resin restorations [1,2]. Discoloration causes color mismatch, consequent patient dissatisfaction, and eventually additional costs for correction/replacement of the restoration.

Discoloration of resin-based materials may be caused by intrinsic or extrinsic factors [2,3]. Extrinsic factors include staining by absorption of colorants as a result of contamination from exogenous sources. Surface discolorations in composite resins are related to hygiene and eating habits, as well as smoking status [4,5]. Red wine, coffee and turmeric are the substances with the greatest pigmenting power, followed by tea, and mate [6,7]. The intrinsic factors involve the discoloration of the resin material itself, such as the alteration of the resin matrix and of the interface of the matrix and the fillers [8]. Chemical discoloration has been attributed to a change or oxidation in the amine accelerator, oxidation in the structure of the polymer matrix, and oxidation of the unreacted pendant methacrylate groups [2,9,10,11]. According to the literature, the color stability of composite resins directly relates to the size, type, and volume of fillers, the type of matrix and monomer, the degree of polymerization, and water absorption [12,13,14,15,16].

Although there is a lot of literature dedicated to the study of the pigmentation of composites [1,2,3,4,5,6,7,8,9,10,11,12,13,14,15,16], the mechanism of discoloration could be explored further. In particular, it is not clear whether the deposition of pigments takes place only on the surface or if it also occurs inside the material and to what extent. However, it would be important to investigate this aspect so that the discoloration can be treated properly.

The aim of this study was to evaluate whether pigments penetrate into composite resins and, if this happens, the extent of pigment penetration. Two composite resins, one nano-filled and one non-homogeneous micro-hybrid were compared. We wanted to observe the behaviour of the resins in vitro and understand if the pigments were deposited only on the surface of the restoration, so as to make it easy to remove them by polishing, or if the deposition also took place inside the material.

## 2. Materials and Methods

Two different commercially available composite resins were compared in the study: a nano-filled composite resin (Filtek Supreme, 3M ESPE, St Paul, MN, USA) and a non-homogeneous micro-hybrid composite resin (Tetric EvoCeram, Ivoclar, Schaan, Liechtenstein) (Table 1). Before carrying out the test, a pilot study was performed, which involved the creation of three specimens for each material, conducted in the same way as the main test was performed. Sample size was determined to highlight a difference between the two composite test resins. To detect a difference between the two composite resins of one effect size considering a difference of 2.8 as being the smallest difference in whiteness magnitude discernible by the human eye [17] with a two-sided 5% significance level (Bonferroni correction α = 0.0036) and a power of 80%, a sample size of 30 specimens per group is necessary.

**Figure 1:**
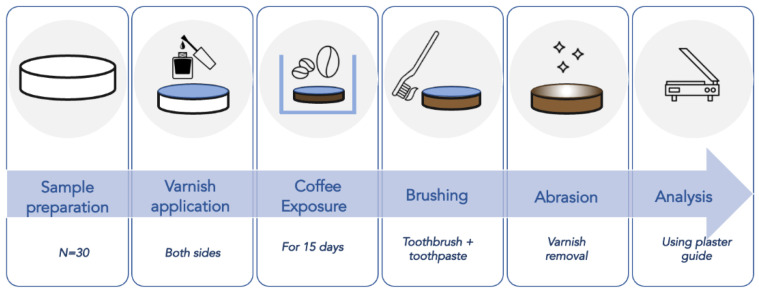
Test groups specimens preparation.

Thirty specimens were made for each of the two materials, ST GROUP: Filtek Supreme Test; ET GROUP: Evo Ceram Test. In addition, two control groups of 10 specimens each were added, SC GROUP: Filtek Supreme Control; EC GROUP: Evo Ceram Control. Figure 1 describes specimens preparation and analysis process.

### 2.1. Specimen Preparation

A cylindrical polypropylene mold with a thickness of 2.0 mm and an internal diameter of 8.4 mm was used for the realization of the specimens. Composite resin was inserted inside the mold until it was filled. Subsequently the mold was enclosed between two glass panes in order to complete the polymerization of the material surface, avoiding the possible inhibition by ambient oxygen, and to obtain smooth and circular surfaces for each specimen. The resin inserted into the mold was cured for 20 s, for each of the two sides, through direct contact between the tip of the light curing unit (Bluephase 10, High Power Program 1200 mW/cm², Ivoclar, Schaan, Liechtenstein). Once the curing process was completed, the glass tops were removed, and the resin specimen was extracted from the polypropylene mold. Any irregularities were eliminated by using abrasive discs (Sof-Lex Pop on 1982 F and SF, 3M ESPE, St Paul, MN, USA). A layer of transparent nail polish (Smart Nail Lacquer, KIKO, Bergamo, Italy) was applied to both flat surfaces of each specimen of composite resin, taking care to leave only the lateral surface free. Thirty specimens of each composite resin were subjected to the pigment treatment. The specimens were immersed in a coffee solution. For the preparation of the solution, 1.6 g of coffee (Nescafè Classic, Nestlè, Vevey, Switzerland) was dissolved in 100 mL of boiling water. The specimens were stored in this solution at 37 °C for 15 days. At the end of the treatment, they were extracted from the solution and washed under tap water for one minute, then the lateral surfaces of the specimens were cleaned with an electric toothbrush (Oral B Pro 600, Procter and Gamble, Cincinnati, OH, USA) and toothpaste (Az Pro Expert, Procter and Gamble, Cincinnati, OH, USA) for about 30 s. The remaining 10 specimens of each group were immersed in physiological solution and stored at 37 °C for 15 days.

### 2.2. Specimen Analysis

At the end of the aging process, each specimen was thinned to a thickness of about 1 mm, acting exclusively on one of the two flat surfaces. A lapping machine (LS2, Remet, Bologna, Italy), on which was mounted an abrasive paper with P320 grain size (WS Flex 18 C, Hermes Abrasives Ltd., Virginia Beach, VA, USA), was used under running water. The achievement of the final thickness of the composite resin disc (1 ± 0.05 mm) was verified using a digital caliper (Micromaster, TESA Technology, Renens, Switzerland).

**Figure 2:**
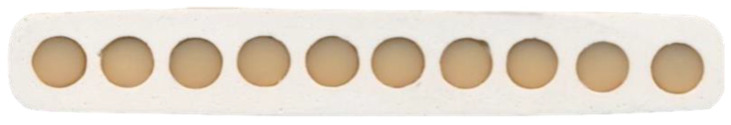
Mold used to analyze and place specimens on the scanner.

The specimens were placed inside a plaster mold that completely enclosed them (Figure 2), leaving only the composite resin surface that had previously been abraded exposed. Subsequently, the plaster mold was positioned in a flatbed scanner (Epson Perfection V850Pro) and a computerized scan (1200 DPI resolution) was started in order to acquire the sectional surface scan of the specimens using photographic software (ADOBE Photoshop CC 2021).

**Figure 3:**
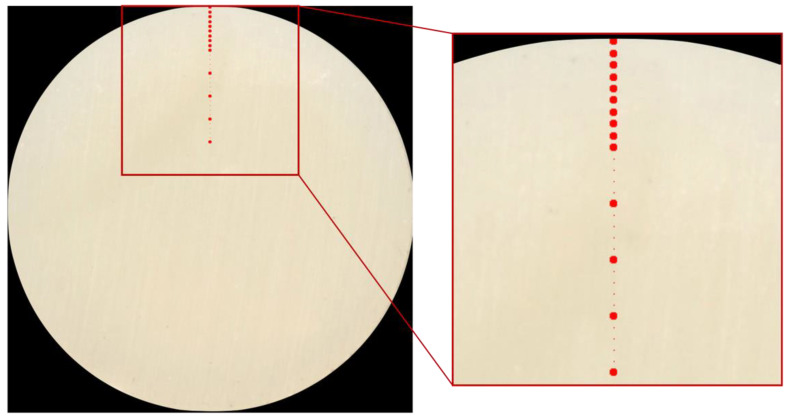
The image shows a schematic representation of different measurement levels.

On each photographic image two lines, perpendicular to each other and passing through the center of the circular surface, were digitally traced. On each line, 14 areas with a size of 25 pixels (5 × 5) were identified (Figure 3). The first at 0.1 mm from the outer perimeter, the following at 0.2 mm, 0.3 mm, 0.4 mm, 0.5 mm, 0.6 mm, 0.7 mm, 0.8 mm, 0.9 mm, 1.0 mm, 1.5 mm, 2.0 mm, 2.5 mm, 3.0 mm from the outer perimeter.

The CIELab system (color notation system of the Commission Internationale de l’Eclairage) was used for the color evaluation. For each area the average values of L* (lightness), a* (redness) and b* (yellowness) were measured, then *W* (whiteness) parameter was calculated. *W* is an alternative whiteness index based on CIELab measurements [18]. This uses a nominal white point, defined as L* = 100, a* = 0 and b* = 0, and calculates the distance the color of a tooth is from in the color space, as follows:

(1) $$W=100-{\left[{\left(a^{*}\right)}^{2}+{\left(b^{*}\right)}^{2}+{\left(100-L^{*}\right)}^{2}\right]}^{\frac{1}{2}}$$

### 2.3. Statistics

Mean and standard deviation were calculated for each group for each measurement depth. The four group were compared using the ANOVA test and the post-hoc Tukey Kramer test. For each statistical model, the residuals were investigated for the assumptions of normality and homoscedasticity.

## 3. Results

**Figure 4:**
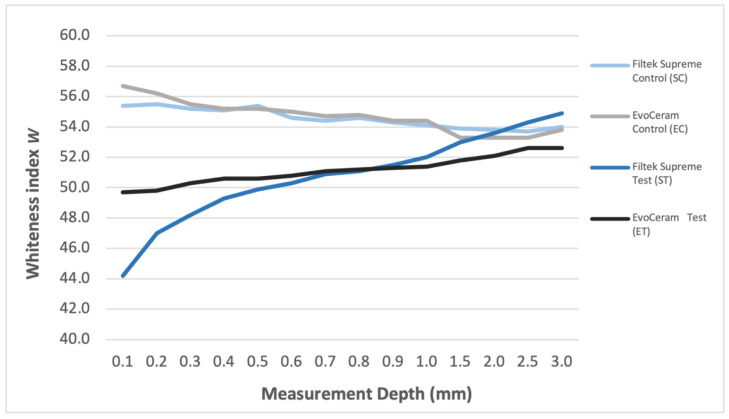
The image shows the trend of *W* for each group. It can be observed that, in the more superficial levels of the specimens, the groups show different values, while, moving towards the center of the specimens, the values tend to become uniform.

The results of the test and statistical analysis are shown in Table 2. The mean and standard deviation of the W value were calculated for each group and for each individual measurement level. The ANOVA t-test demonstrated a statistically significant difference (*p* < 0.0001) between test and control groups up to a depth of 1.0 mm for the Supreme XTE composite and up to a depth of 2.0 mm for the EvoCeram composite and at 3.0 mm depth for both composites. Statistically significant differences were also found between Test groups. The Tukey-Kramer test showed a different type of behavior of the specimens prepared with the two composite resins when immersed in the pigmenting solution up to a depth of 0.3 mm favoring ET group and after a depth of 1.5 mm favoring ST group. Figure 4 shows how the value of W changes between the various groups, in the various areas of the specimen. In the innermost layers the values tend to become uniform. No differences were observed between the two control groups.

## 4. Discussion

This study examined 80 specimens, made with two different types of composite resins. Test groups were subjected to an aging treatment, by immersion in a soluble coffee solution at 37 °C for 15 days. Control groups specimens were immersed in physiologic solution at 37 °C for the duration of the test. Considering that 24 h of in vitro immersion correspond to approximately one month of in vivo aging [7], it can be assumed that the specimens have undergone aging comparable to a year of clinical service.

Two different commercially available composite resins were compared in the study: a nano-filled composite resin (Filtek Supreme, 3M ESPE, St Paul, MN, USA) and a non-homogeneous micro-hybrid composite resin (Tetric EvoCeram, Ivoclar, Schaan, Liechtenstein) (Table 1). The chosen composites are widely used, both in the anterior and posterior sector. Filtek Supreme is a nano-hybrid composite, where the largest filler particles are composed of nanoparticles clusters [19]. The individual particles of the cluster are not individually silanized [19]. EvoCeram is a microhybrid composite where a part of the filler is made up of a prepolymerized composite, subsequently fragmented [20]. Again, the prepolymerized fragments are not silanized. The lack of a complete silanization of the matrix/filler interface could lead to a greater possibility of infiltration of water [21] and therefore of pigments.

The specimens measure 2.0 mm of thickness and 8.4 mm of diameter in order to ensure that their polymerization was homogeneous and complete. In fact, 2.0 mm of thickness is the maximum limit to obtain a uniform polymerization of the whole material [22,23,24]. A correct polymerization is the basis of the good aesthetic and mechanical properties of composite resins. On the contrary, an incomplete polymerization of the material can lead to the absorption of water and exogenous substances that can be responsible for a progressive deterioration of the material [7]. As far as the diameter of the specimens is concerned, its measurement corresponds to the size of the tip of the curing light, thus allowing to obtain a polymerization in a single phase.

The lateral surface of each specimen of composite resin was subjected to polishing using abrasive discs (Sof-Lex Pop-on 1982 F and SF, 3M ESPE, St Paul, MN, USA) in order to obtain a smooth surface, and therefore non-retentive for chromogenic substances, a layer of transparent nail polish was applied to the flat surfaces of the discs, in order to isolate the upper and lower part of the disc, ensuring that the food coloring only penetrated from the lateral surface.

From the literature it emerged that the substances with greater pigmenting power are coffee, red wine and turmeric [6,7,25]. The decision to immerse the test specimens in coffee was due to the fact that it is consumed in large quantities and daily all over the world. Coffee contains very small yellow molecules with coloring power which appear to be responsible for the problem of color change in composites due to their affinity with the structure of polymers [6,7,26].

Brushing the lateral surface of the resin discs with an electric toothbrush and toothpaste, following the aging procedure, had the purpose of eliminating any extrinsic pigmentations adhering to the surface that could have influenced the color measurement [27,28]. All the specimens thus obtained were placed on the glass plate of a flatbed scanner (Epson Perfection V850Pro) and a computerized scan was started in order to acquire, using photographic software (ADOBE Photoshop CC 2021), the scan of the sectional surface of the specimens. To avoid that ambient light and the light emitted by the scanner itself could create shadow areas that could affect the measurement, a white plaster mold was created in which to insert the specimen before scanning was started.

The results showed a statistically significant difference between the four groups. The two composite resins immersed in the physiological solution have similar trend in *W* values and have not undergone color variations at any measurement level. This seems to confirm that in the absence of chromogenic substances, composite resins tend to maintain their original color for the storage time.

Filtek Supreme composite resin when subjected to the pigmenting treatment underwent a statistically significant color change up to 1 mm in depth, compared to the same resin stored in the absence of chromogenic substance. The EvoCeram composite resin, on the other hand, underwent a statistically significant change until 2 mm. Although, compared to Filtek Supreme, the color change was less marked in the surface layers. From Figure 4 it can be observed a different trend in the two composites as regards the penetration of pigments inside the material. Examining the slope of the *W* trend line of the EvoCeram composite, it can be observed that this has a more constant trend than the Filtek Supreme composite.

Filtek Supreme, accordingly to other studies [16,29], seems to be more subjected to staining, probably due to its structure that presents nano-aggregated particles. These structures have an interface that is not perfectly silanized. This can lead to the infiltration of water and pigments.

The present study uses the parameter *W* (white index) to determine the degree of pigmentation. A whiteness index was chosen, as the future aim is to subject the previously pigmented samples to whitening procedures. Moreover, the advantage of using *W* is precisely that of having an absolute index, which compares the measured color with a defined value. In the literature there are innumerable indices that attest to the changes in color or the white index itself. Luo et al. [30] claim that all indices (*WIC*, *WIO* and *W*), *b* * and Δ*E* values showed a similar trend; they are equally good in determining the color change.

When the pigmentation of the composite becomes clinically unacceptable, then comes the need to restore the initial color. One solution would be to remove the surface layer of the restoration and lay new material over the restoration. Otherwise, it is necessary to replace the entire restoration. The possibility of restoring the initial color of the resins through the application of bleaching agents is very interesting. However, in the literature, there is a small number of studies that demonstrate its effectiveness [31,32]. According to these studies it would seem that peroxides are able to whiten these materials; however, this occurs only in the superficial layers. There are no studies whose objective is to analyze the behavior of whitening agents in depth.

Some limitations have to be considered due to the nature of the study. A possible error could emerge from the analysis of the specimens using a scanner. In fact, by analyzing the results it can be seen that even the control groups have the whiter external levels while the internal part has a higher *W* value. However, the differences are not statistically significant. A possible explanation could be given by the presence of the white plaster mold which can reflect the light, influencing the color of the outermost parts of the specimen, during the measurement. In any case, this would be a systematic error which affects equally the test and control groups.

## 5. Conclusions

Within the limitations of this in vitro study, the following conclusions were drawn. The two composite resin materials, subjected to pigmenting treatment, underwent a color variation with different patterns. It emerged that the Filtek Supreme composite proved to be more sensitive to color change than the EvoCeram composite in the surface. However, the pigmentation process seemed to go deeper for the EvoCeram composite.

## Figures and Tables

**Table 1:** List of tested materials.

Commercial Nameand Manufacturer	Filler Content (vol%)	Matrix Composition	Filler Composition
Filtek Supreme XTE (3M ESPE)	63	Bis-GMA, UDMA, TEGDMA, PEGDMA, Bis-EMA.	Combination of non-agglomerated/non-aggregated 20-nm silica filler, non-agglomerated/non-aggregated 4 to 11 nm zirconia filler, and aggregated zirconia/silica cluster filler.
Tetric EvoCeram (Ivoclar)	53–55	UDMA, Bis-GMA, ethoxylated Bis-EMA.	Inorganic fillers (40 nm–3 μm): barium glass, ytterbium trifluoride, mixed oxide (SiO_2_/ZrO_2_).

**Table 2:** Mean (and standard deviation) of *W* are listed. Statistical differences are represented by letters. Different letters indicate a significant difference according to the Tukey-Kramer test.

Measurement Depth	SC (N = 10)	EC (N = 10)	ST (N = 30)	ET (N = 30)	*p*-Value
0.1 mm	55.4 (1.5) ^a^	56.7 (1.7) ^a^	44.2 (4.5) ^b^	49.7 (3.9) ^c^	<0.0001
0.2 mm	55.5 (1.1) ^a^	56.2 (1.5) ^a^	47.0 (3.0) ^b^	49.8 (2.7) ^c^	<0.0001
0.3 mm	55.2 (1.2) ^a^	55.5 (1.2) ^a^	48.2 (2.6) ^b^	50.3 (2.4) ^c^	<0.0001
0.4 mm	55.1 (1.2) ^a^	55.2 (0.9) ^a^	49.3 (2.1) ^b^	50.6 (2.0) ^b^	<0.0001
0.5 mm	55.4 (1.4) ^a^	55.2 (1.0) ^a^	49.9 (2.0) ^b^	50.6 (1.9) ^b^	<0.0001
0.6 mm	54.6 (1.5) ^a^	55.0 (1.1) ^a^	50.3 (1.5) ^b^	50.8 (1.7) ^b^	<0.0001
0.7 mm	54.4 (0.9) ^a^	54.7 (1.1) ^a^	50.9 (1.4) ^b^	51.1 (1.7) ^b^	<0.0001
0.8 mm	54.6 (1.0) ^a^	54.8 (1.5) ^a^	51.1 (1.2) ^b^	51.2 (1.5) ^b^	<0.0001
0.9 mm	54.3 (1.3) ^a^	54.4 (0.9) ^a^	51.5 (1.3) ^b^	51.3 (1.3) ^b^	<0.0001
1.0 mm	54.1 (1.1) ^a^	54.4 (1.0) ^a^	52.0 (1.2) ^b^	51.4 (1.2) ^b^	<0.0001
1.5 mm	53.9 (0.8) ^a^	53.3 (1.3) ^a^	53.0 (1.2) ^a^	51.8 (1.6) ^b^	<0.0001
2.0 mm	53.8 (1.0) ^a^	53.3 (1.0) ^a^	53.6 (1.0) ^a^	52.1 (1.4) ^b^	<0.0001
2.5 mm	53.7 (0.7) ^a,b^	53.3 (0.8) ^a,b^	54.3 (1.0) ^a^	52.6 (1.5) ^b^	<0.0001
3.0 mm	54.0 (0.7) ^a^	53.8 (0.8) ^a^	54.9 (1.0) ^b^	52.6 (1.3) ^c^	<0.0001

SC, Filtek Supreme Control; EC, EvoCeram Control; ST, Filtek Supreme Test; ET, EvoCeram Test.
